# Nebulized dexmedetomidine for attenuating hemodynamic response to laryngoscopy and endotracheal intubation in adult patients undergoing surgeries under general anaesthesia: a systematic review and meta-analysis of randomized controlled trials

**DOI:** 10.1186/s12871-023-02366-9

**Published:** 2023-12-11

**Authors:** Mayank Gupta, Rachna Rohilla, Priyanka Gupta, Hemanthkumar Tamilchelvan, Udita Joshi, Jyoti Kanwat

**Affiliations:** 1 Department of Anaesthesiology, All India Institute of Medical Sciences, Bathinda, Punjab India; 2 Department of Pharmacology, All India Institute of Medical Sciences, Bathinda, India; 3 Department of Anaesthesiology, Graphic Era Institute of Medical Sciences, Dehradun, Uttarakhand India; 4 Banglore Hospice Trust, Bengaluru, Karnataka India

**Keywords:** Dexmedetomidine, Endo-tracheal intubation, Laryngoscopy, Meta-analysis, Systematic review, Hemodynamic response, Stress response

## Abstract

**Background:**

Sympathetic stimulation associated with laryngoscopy and endotracheal intubation (ETI) may lead to adverse cardio-/cerebro-vascular events in susceptible patients. Nebulization is a novel route for dexmedetomidine administration providing a large surface area for absorption while avoiding bradycardia and hypotension associated with intravenous route. We aimed to evaluate the efficacy and safety of dexmedetomidine nebulization for attenuating hemodynamic response to ETI in adult patients undergoing surgery under general anaesthesia.

**Methods:**

This systematic review was registered prospectively in the International Prospective Register of Systematic Reviews (CRD42023403624). PubMed, Embase (OvidSP), Cochrane library, Scopus (Elsevier), Web of Science (Clarivate) and Google Scholar were systematically searched from database inception until March 31, 2023. Two reviewers independently screened titles, abstracts and then full text against pre-specified eligibility criteria. Randomized controlled trials (RCTs) assessing effect of dexmedetomidine nebulization on hemodynamic response to ETI in adult patients undergoing surgeries under general anaesthesia were included. All studies reporting heart rate and systolic blood pressure at baseline and various time points after ETI were included. A pre-piloted data extraction form, Cochrane revised risk-of-bias tool (ROB 2) tool, GRADE approach and RevMan 5.4.1 (Cochrane Collaboration, Copenhagen, Denmark) were used for data extraction, risk of bias assessment, rating certainty of evidence and data synthesis respectively. Mean difference and relative risk with 95% Confidence Interval (CI) were used for continuous and dichotomous variables respectively.

**Results:**

Six RCTs randomized 480 patients with ASA I/II patients aged < 60 years of age and undergoing elective surgeries to receive either dexmedetomidine (*n* = 240) or saline nebulization (*n* = 240). Except for one RCT which used 2 μg/kg, all other RCTs used dexmedetomidine dose of 1 μg /kg. Heart rate, systolic, diastolic and mean blood pressure were significantly lower in the dexmedetomidine group at all the measured time points after laryngoscopy and ETI with the only exception being systolic blood pressure at 3 min [mean difference -13.86 (95% CI -30.01 to 2.99), *p* = 0.09]. Bradycardia and hypotension as adverse effects were absent across the included studies. However, only one-third of the included studies had a low risk of bias and strength of evidence was very low according to the GRADE assessment.

**Conclusions:**

Compared to placebo, premedication with dexmedetomidine nebulization was associated with lower HR and BP following ETI without any risk of bradycardia and hypotension. However, the strength of evidence was very poor and came from just one country. Future well designed and conducted studies in different populations are warranted.

**Trial registration:**

PROSPERO Registration number: CRD42023403624

**Supplementary Information:**

The online version contains supplementary material available at 10.1186/s12871-023-02366-9.

## Background

Laryngoscopy and endotracheal intubation (ETI) are associated with sympathetic stimulation induced hemodynamic changes [1]. The consequent increase in heart rate (HR) and blood pressure (BP) though short-lived may lead to myocardial infarction, cardiac arrhythmias, cardiac failure and cerebrovascular accidents in patients with underlying cardiovascular or cerebrovascular diseases [1]. Premedication with various agents has been shown to attenuate this sympathetic response and its associated risk of arrhythmias and myocardial infarction [1]. However, none of them is ideal and each of them is associated with its unique adverse effects like hypotension, bradycardia, chest rigidity or increased bronchomotor tone [1].

Dexmedetomidine is a centrally acting α-2 adrenergic agonist with sedative, hypnotic, analgesic, anxiolytic, antisialagogue, antinociceptive and sympatholytic action [2, 3]. Premedication with dexmedetomidine through intravenous, intramuscular and intranasal route has been shown to effectively attenuate hemodynamic response to laryngoscopy and ETI [4–10]. However, its use is associated with adverse effects like hypotension and bradycardia with intravenous route and nasal irritation with intranasal route [5–8, 11]. A 2021 systematic review and meta-analysis (SRMA) showed intravenous dexmedetomidine to significantly attenuate tracheal intubation associated increase in HR and BP but associated with significant risk of bradycardia and hypotension; recommending cautious evaluation while using it in daily practice [4].

Nebulization provides an alternative route of dexmedetomidine premedication with high bioavailability through both nasal (65%) and oral mucosa (82%) and avoid a venipuncture as a prerequisite. Recent studies have shown nebulisation as a novel route of dexmedetomidine administration for attenuation of hemodynamic response to ETI [12–14]. However, no current or planned systematic review evaluating the safety and efficacy of nebulized dexmedetomidine for blunting hemodynamic response to laryngoscopy and ETI in adult patients was identified.

Therefore, the present SRMA was conducted to systematically identify, collate, critically appraise and synthesize available evidence on dexmedetomidine nebulisation for attenuating hemodynamic response to laryngoscopy and ETI in adult patients (≥ 18 years) undergoing surgery under general anaesthesia. Our findings will help clinicians in evidence based decision-making and formulation of institutional guidelines.

## Methods

This systematic review has been reported in accordance with the Preferred Reporting Items for Systematic reviews and Meta-analysis (PRISMA) 2020 standards [15]. The protocol for this systematic review was registered prospectively in the International Prospective Register of Systematic Reviews (PROSPERO No. CRD42023403624).

### Search strategy

A preliminary search helped in identifying thesaurus and free text terms for the key concepts (laryngoscopy, endotracheal intubation and dexmedetomidine). The thesaurus and free text terms for a similar concept were combined using Boolean operator “OR”. The search strings for different concepts were then combined using Boolean operator “AND”. A systematic and comprehensive literature search was performed in the following electronic bibliographic databases: PubMed, Embase and Embase Classic (OvidSP), Cochrane library (https://www.cochranelibrary.com), Scopus (Elsevier), Web of Science (Clarivate) and Google Scholar from inception to 31st March 2023. The search strategy was limited to randomized controlled trials (RCTs) conducted in humans and published in English language. No date or age restrictions were applied. The search strategy was first formulated for Embase and later adapted for other databases. The search strategy and literature searches were formulated and conducted by one reviewer (MG) and re-ran by another experienced reviewer (UJ) to rule out syntax or any other error. The search strings for all the databases as they were run has been reported in the Supplementary file 1.

To ensure literature saturation, reference lists of included articles and relevant systematic reviews were screened and citation tracking of included articles (on Google scholar and Scopus) was done to identify any additional relevant article. Screening of reference lists and citation tracking continued until no new articles were identified.

### Study selection

PICOS format helped operationalise the review question into key inclusion and exclusion criteria. RCTs comparing preoperative administration of dexmedetomidine nebulization with either placebo or no intervention for attenuating hemodynamic response (as measured by HR and BP at any time point up to 10 min after ETI) to laryngoscopy and ETI in adult patients (≥ 18 years of age) undergoing surgery under general anaesthesia with ETI were included. Studies evaluating dexmedetomidine nebulization in pediatric patients, adult patients undergoing tracheal intubation other than for surgery or dexmedetomidine administration through any other route were excluded. Non-randomized studies and evidence synthesis were not included, however, their reference lists were screened to identify any eligible study missed through database searching.

Studies identified through database search were transferred to EndNote reference manager software (V.20, Clarivate Analytics, Philadelphia, Pennsylvania, USA) for de-duplication. De-duplicated results were transferred to Rayyan (www.rayyan.ai) in which two reviewers (MG, HT) independently performed title and abstract screening against the pre-defined inclusion and exclusion criteria (Table 1). Full texts of potentially eligible studies and studies where eligibility could not be determined from title/abstract screening were retrieved and screened independently by two reviewers (MG, HT) for inclusion. Any discrepancies were resolved through discussion and arbitrated by a third reviewer (PG) where necessary. An audit trail of all the disagreements, reasons for the same and resolutions made ensured trustworthiness of the process.

**Table 1:** Characteristics of RCTs included in meta-analysis

Author Year, Country	Pooled age (Mean ± SD)	Males (N)	Intervention		Control		Induction regimen	Results		
			Dexmed	N	Placebo	N		Hemodynamic	Adverse effects	Other
Hussain 2019, India [16]	34.5 ± 5.23	31	Dexmed 2 μg /kg in 5 ml NS 30 min before induction and laryngoscopy	35	5 ml NS	35	Fentanyl 2 μg /kg, Propofol 2 mg/kg, Vecuronium 0.1 mg/kg IV	Control group had statistically higher values of HR, SBP, DBP, MAP after intubation at all time intervals compared to dexmedetomidine group.	No adverse event reported in either group	
Misra 2021, India [12]	39.15 ± 11.25	72	Dexmed 1 μg /kg in 3–4 ml NS 30 min before induction and laryngoscopy	60	3–4 ml NS	60	Ondansetron 4 mg, Midazolam 1 mg, Fentanyl 2 μg g/kg, propofol 10 mg boluses, vecuronium 0.15 mg/kg IV	After laryngoscopy and intubation, a significant trend of decrease in HR in dexmedetomidine group versus control group at all time points. No difference in SBP response between two groups.	Post-operative nausea and vomiting in 3/57 patients in dexmedetomidine group and 1/59 in normal saline group.	Induction dose of propofol, intraoperative fentanyl consumption and mean isoflurane requirements were significantly less in dexmedetomidine group versus control group.
Sheth 2021, India [17]	29 ± 7.52	27	Dexmed 1 μg /kg in 5 ml NS 10 min before shifting to OT	25	5 ml NS	25	Glycopyrrolate 0.005 mg/kg IM, Fentanyl 2 μg/kg, Propofol 2 -2.5 mg/kg, atracurium 0.5 mg/kg IV	There was significantly reduced SBP, DBP, MAP at 1, 5 and 10 min after laryngoscopy and intubation in dexmedetomidine compared to control group. No difference between the two groups in terms of HR response.	No adverse event reported in either group	
Suryawanshi 2022, India [18]	43.41 ± 13.21	31	Dexmed 1 μg /kg in 3–4 ml NS 30 min before induction and laryngoscopy	30	3–4 ml NS	30	Glycopyrrolate 0.004 mg/kg IV, Inj. Midazolam 0.02 mg/kg IV and Inj. Fentanyl 1- 2 μg /kg IV, propofol 2 mg/kg, Succinylcholine 2 μg/kg IV	HR, SBP, DBP, MAP significantly lower in dexmedetomidine group at all time points studied as compared to the control group.	No adverse event reported in either group	Lower post-operative sore throat in dexmedetomidine group.
Shrivastava 2022, India [19]	38.97 ± 14.19	28	Dexmed 1 μg /kg in 5 ml NS 30 min before induction and laryngoscopy	50	5 ml NS	50	Alprazolam 0.5 mg Midazolam 1 mg, Fentanyl 2 μg/kg, propofol 1–2 mg/kg, Vecuronium 0.1 mg/kg IV	SBP, DBP, MAP and HR significantly lower in dexmedetomidine group compared to control group at 1, 5, 10 min.	No adverse event reported in either group	Nebulised dexmedetomidine has a dose sparing effect on induction dose of propofol.
Kaila 2023, India [20]	35.43 ± 7.97	43	Dexmed 1 μg/kg in 3–4 ml NS 30 min before induction and laryngoscopy	40	3–4 ml NS	40	Metoclopramide 10 mg IV, Ondansetron 8 mg, Fentanyl 1–2 μg/kg, Propofol 2 mg/kg, Succinylcholine 100 μg IV	SBP, DBP, MAP significantly lower in dexmedetomidine group compared to control group at 2,4,6,8 min.	No adverse event reported in either group	

*HR* Heart Rate, *DBP* Diastolic Blood Pressure, *IV* Intravenous, *Kg* Kilogram, *MBP* Mean Blood Pressure, *mg* Milligram, *μg* Microgram, *SBP* Systolic Blood Pressure

### Outcome measures

The primary outcome measures analyzed were heart rate (HR) and systolic blood pressure (SBP) at baseline and at any time point till 10 min after ETI (as provided in the published report). Other additional outcomes included diastolic (DBP) and mean (MBP) blood pressure at any time point till 10 min after ETI. We also collected data on bradycardia (% of patients), hypotension (% of patients) and postoperative nausea (% of patients), vomiting (% of patients), respiratory depression (% of patients) or any other adverse outcome as reported in the study.

### Data extraction

Two reviewers (MG and RR) independently extracted following data from included studies on a pre-piloted data extraction excel form designed specifically for this review: first author, publication year, inclusion and exclusion criteria, details of intervention and control group (dose, mode of nebulisation, duration of administration), number and demographic characteristics of participants in each group, surgical procedures, rate and reasons for dropout and outcome parameters. Following outcome parameters were retrieved: HR, SBP, DBP and MBP at baseline and at all time till 10 min (as provided in the published report) after ETI; drugs used at induction of anaesthesia and their doses, intraoperative bradycardia or hypotension (% patients) and postoperative nausea and vomiting (% patients). Corresponding authors of included studies were contacted through e-mail for any missing data. Any discrepancies between the reviewers in the extracted data were resolved through discussion.

### Risk of bias assessment and rating certainty of evidence

Two reviewers (MG and RR) independently assessed each included study for risk of bias using Cochrane revised risk-of-bias tool for randomized trials (RoB 2) [21, 22]. RoB-2 assessment was done using the RoB Excel Tool (https://www.riskofbias.info/welcome/rob-2-0-tool/current-version-of-rob-2). The tool assesses each study for risk of bias on five domains: risk of bias arising from the randomization process, bias arising due to deviations from the intended interventions, bias arising due to missing outcome data, bias in measurement of outcome and bias in selection of the reported result. Each domain in individual studies was graded as “low risk of bias”, “some concerns” or “high risk of bias” for each included study graded across all domains [21]. The overall risk of bias for individual studies was determined by highest RoB level in any domain. Any discrepancies were resolved by discussion and arbitrated by a third reviewer (PG) where necessary.

Two authors (MG and RR) independently rated the certainty of evidence according to the Grading of Recommendations, Assessment, Development and Evaluation (GRADE) working group system using the GRADEpro software (https://www.gradepro.org). The quality of evidence was downgraded depending upon the degree of bias, inconsistency, indirectness and imprecision. Any disagreements were resolved through consultation and arbitrated by a third author if required (PG).

### Statistical analysis

We performed inverse variance random-effect meta-analysis using RevMan 5.4.1 [Review Manager Version 5.4.1, The Cochrane Collaboration, 2020]. HR, SBP, DBP and MBP were treated as continuous variables and incidence of adverse effects (bradycardia, hypotension) as dichotomous variables. Continuous outcomes were reported as weighted mean difference with 95% Confidence Interval (CI). Significance was set at *P* < 0.05. Outcome heterogeneity between the studies was assessed using Cochran’s Q test and quantified with I-square statistic. I-square > 50% was considered as statistically significant heterogeneity between the studies. Sensitivity analysis was performed by removing studies using different doses of nebulized dexmedetomidine and observing its effect on outcome heterogeneity and effect estimate. Subgroup analysis was performed if there were more than equal to three studies using different doses of nebulized dexmedetomidine.

## Results

### Search results

**Fig. 1:**
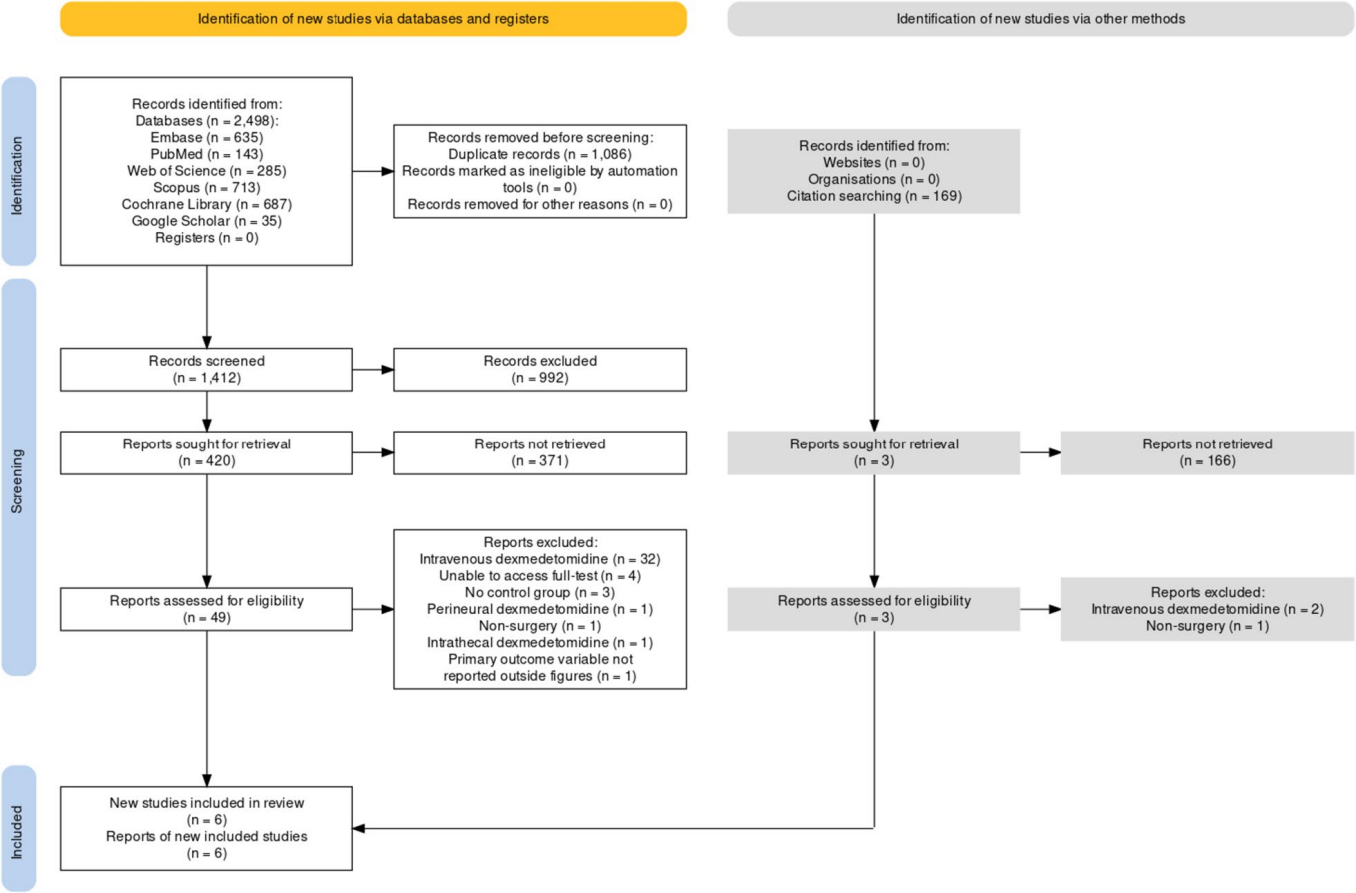
PRISMA flow diagram

Database search identified 1412 records after excluding 1082 duplicates. Forty-nine reports underwent full-text screening after removing 1363 records during title and abstract screening. No new articles were identified through reference list screening and citation tracking. We excluded 43 reports for reasons cited in PRISMA flow diagram created using Shinny app (Fig. 1) [23]. A total of seven studies were selected [12, 13, 16–20]. However, Kumar et al. did not mention effect on HR which was the primary outcome of the study and data for BP (SBP, DBP and MBP) was provided only in graphical figures in the article from which the exact mean (SD) values at each time point could not be extracted for meta-analysis [13]. The data could not be obtained despite email request and hence the study was excluded from the data-synthesis [13]. A total of six randomized controlled trials underwent quality appraisal and data synthesis [12, 16–20].

### Study characteristics

Six studies randomized 480 American society of Anaesthesiologist (ASA) I and/or II patients, < 60 years of age undergoing elective surgeries into intervention (*n* = 240) and control group (*n* = 240) [12, 16–20]. The dose of dexmedetomidine used was 1 mcg/kg in all except one study which used 2mcg/kg (Table 1). All used normal saline as placebo in the control group. The main source of clinical heterogeneity was due to the anaesthesia regimen (Table 1). All reported stress response to endotracheal intubation as the primary outcome. However, only three studies reported sample size calculation [12, 17, 19]. The primary outcome of the studies were effect on HR, SBP and secondary outcomes were effect on DBP, MAP and safety analysis.

### Synthesis of results

All the six studies mentioned above were included in the meta-analysis for primary outcome using random effect model. The data on each time point was not provided by all the studies included. So the analysis for each time point included those studies which provided data for that time point.

#### Primary outcomes

##### Effect on heart rate (HR)

**Fig. 2:**
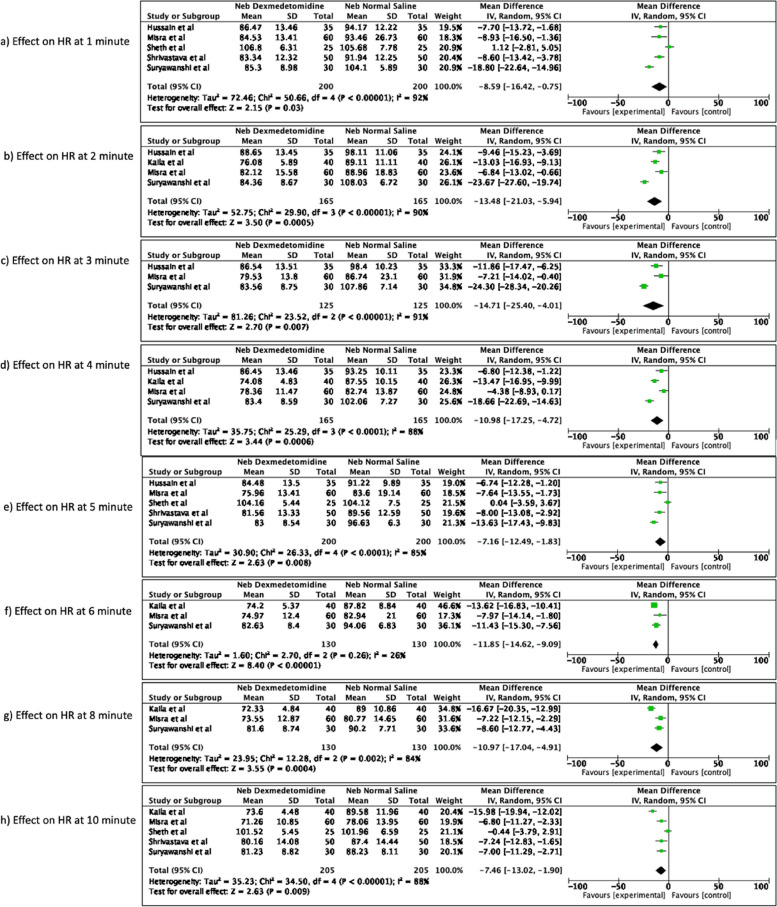
Effect of nebulized dexmedetomidine on heart rate

The nebulized dexmedetomidine significantly reduced the mean HR as compared to the control group at all measured time points [mean difference -8.59 (95% CI -16.42 to -0.75), *p* = 0.03, I^2^ = 92% at 1 min (Fig. 2a); mean difference -13.48 (95% CI -21.03 to -5.94), *p* = 0.0005, I^2^ = 90% at 2 min (Fig. 2b); mean difference -14.71 (95% CI -25.40 to -4.01); *p* = 0.007, I^2^ = 91% at 3 min (Fig. 2c); mean difference -10.98 (95% CI -17.25 to -4.72), *p* = 0.0006, I^2^ = 88% at 4 min (Fig. 2d); mean difference -7.16 (95% CI -12.49 to -1.83), *p* = 0.008, I^2^ = 85% at 5 min (Fig. 2e); mean difference -11.85 (95% CI -14.62 to -9.09), *p* < 0.00001, I^2^ = 26% at 6 min (Fig. 2f); mean difference -10.97 (95% CI -17.04 to -4.91), *p* = 0.0004, I^2^ = 84% at 8 min (Fig. 2g) and mean difference -7.46 (95% CI -13.02 to -1.90), *p* = 0.009, I^2^ = 88% at 10 min (Fig. 2h)] of endotracheal intubation. However there was high heterogeneity in the studies included with I^2^ > 84% at all the time points, except at 6 min where heterogeneity was 26%.

##### Effect of on SBP

**Fig. 3:**
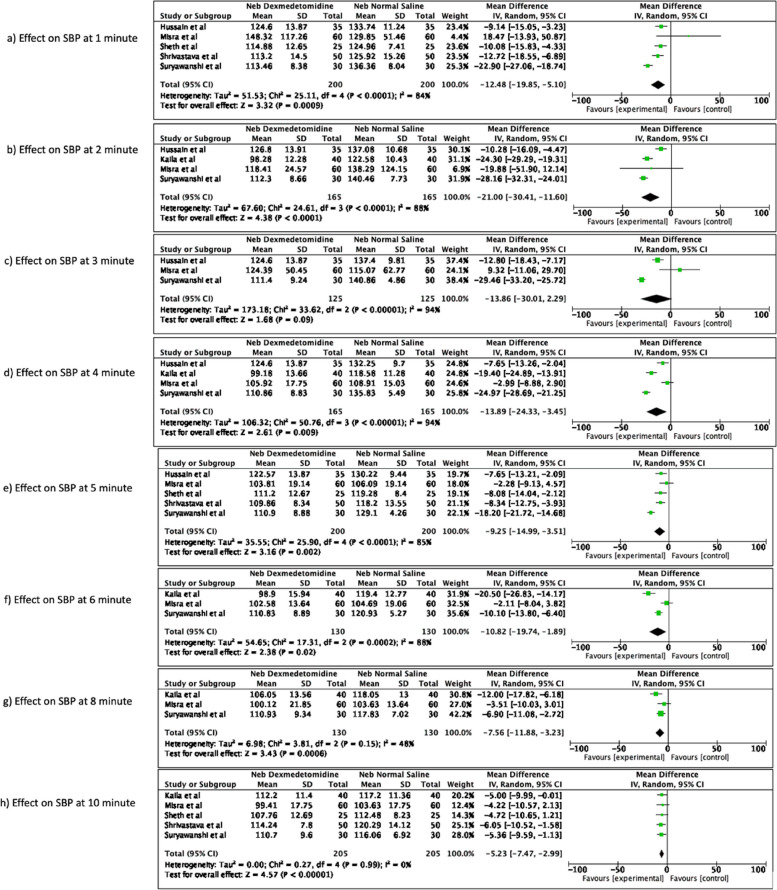
Effect of nebulized dexmedetomidine on systolic blood pressure

The nebulized dexmedetomidine significantly reduced the mean SBP as compared to normal saline at all measured timepoints [mean difference -12.48 (95% CI -19.85 to -5.10), *p* = 0.0009, I^2^ = 84% at 1 min (Fig. 3a); mean difference -21.00 (95% CI -30.41 to -11.60), *p* < 0.0001, I^2^ = 88% at 2 min (Fig. 3b); mean difference -13.89 (95% CI -24.33 to -3.45), *p* = 0.009, I^2^ = 94% minute at 4 min (Fig. 3d); mean difference -9.25 (95% CI -14.99 to -3.51), *p* = 0.002, I^2^ = 85% at 5 min (Fig. 3e); mean difference -10.82 (95% CI -19.74 to -1.89), *p* = 0.02, I^2^ = 88% at 6 min (Fig. 3f); mean difference -7.56 (95% CI -11.88 to -3.23), *p* = 0.0006, I^2^ = 48% at 8 min (Fig. 3g); and mean difference -5.23 (95% CI -7.47 to -2.99), *p* < 0.00001, I^2^ = 0% at 10 min (Fig. 3h)] after endotracheal intubation, except at 3 min after intubation where although overall reduction in SBP was observed with nebulized dexmedetomidine as compared to nebulized normal saline but was not statistically significant [mean difference -13.86 (95% CI -30.01 to 2.99), *p* = 0.09, I^2^ = 94%] as the overall effect 95% CI crosses the line of no difference (Fig. 3c).

#### Secondary outcomes

The secondary end point was effect of nebulized dexmedetomidine on DBP and MAP as compared to nebulized normal saline at various end points.

##### Effect on DBP

**Fig. 4:**
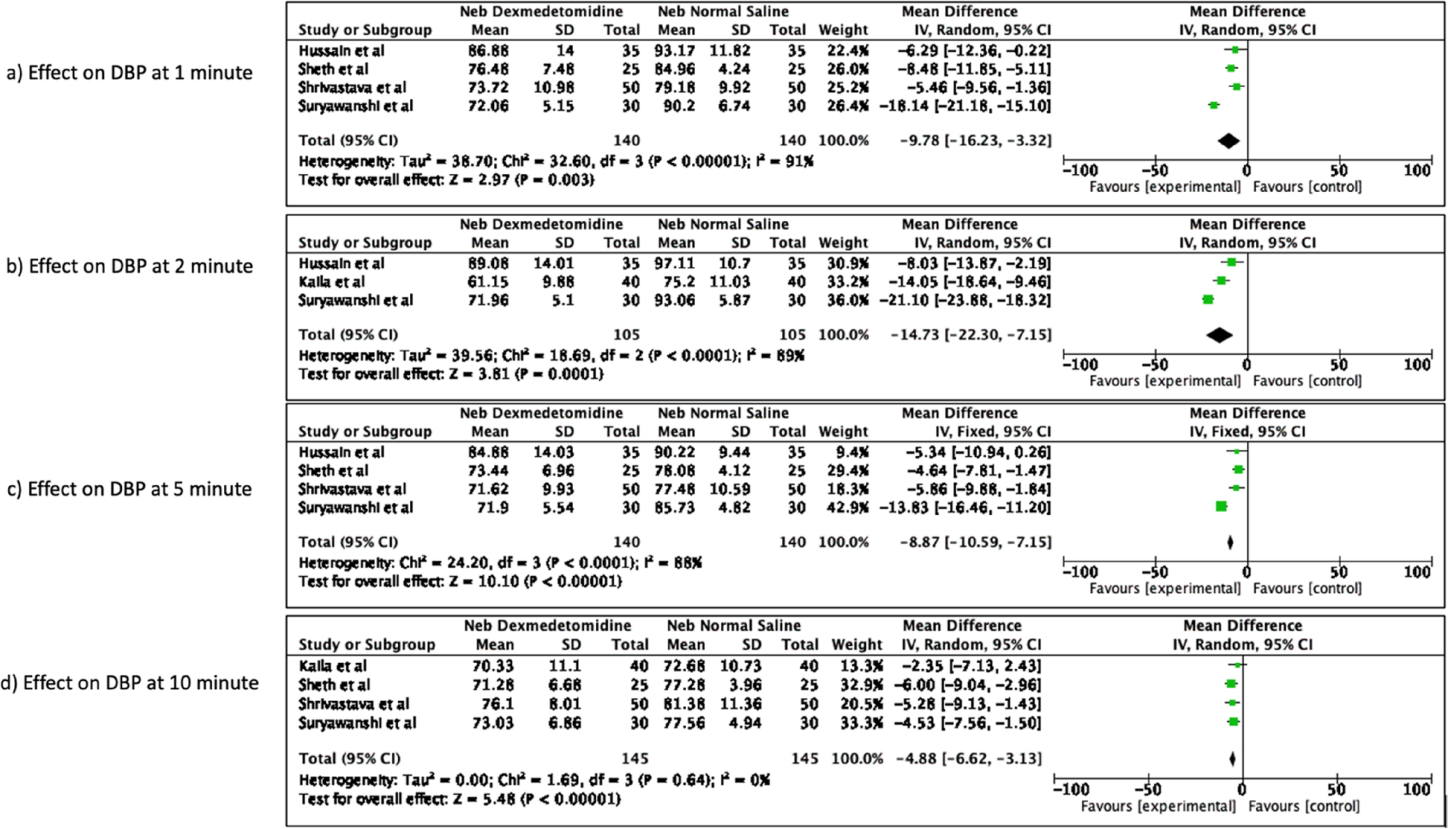
Effect of nebulized dexmedetomidine on DBP

The nebulized dexmedetomidine as compared to normal saline significantly reduced the mean DBP at 1 min after endotracheal intubation and the reduction in DBP persisted till 10 min [mean difference -9.78 (95% CI -16.23 to -3.32), *p* = 0.003, I^2^ of 91% at 1 min; mean difference -14.73 (95% CI -22.30 to -7.15), *p* = 0.0001, I^2^ = 89% at 2 min; mean difference -8.87 (95% CI -10.59 to -7.15), *p* < 0.00001, I^2^ = 88% at 5 min; mean difference -4.88 (95% CI -6.62 to -3.13), *p* < 0.00001, I^2^ = 0% at 10 min post-intubation]. The forest plots are available as Fig. 4.

##### Effect on MBP

**Fig. 5:**
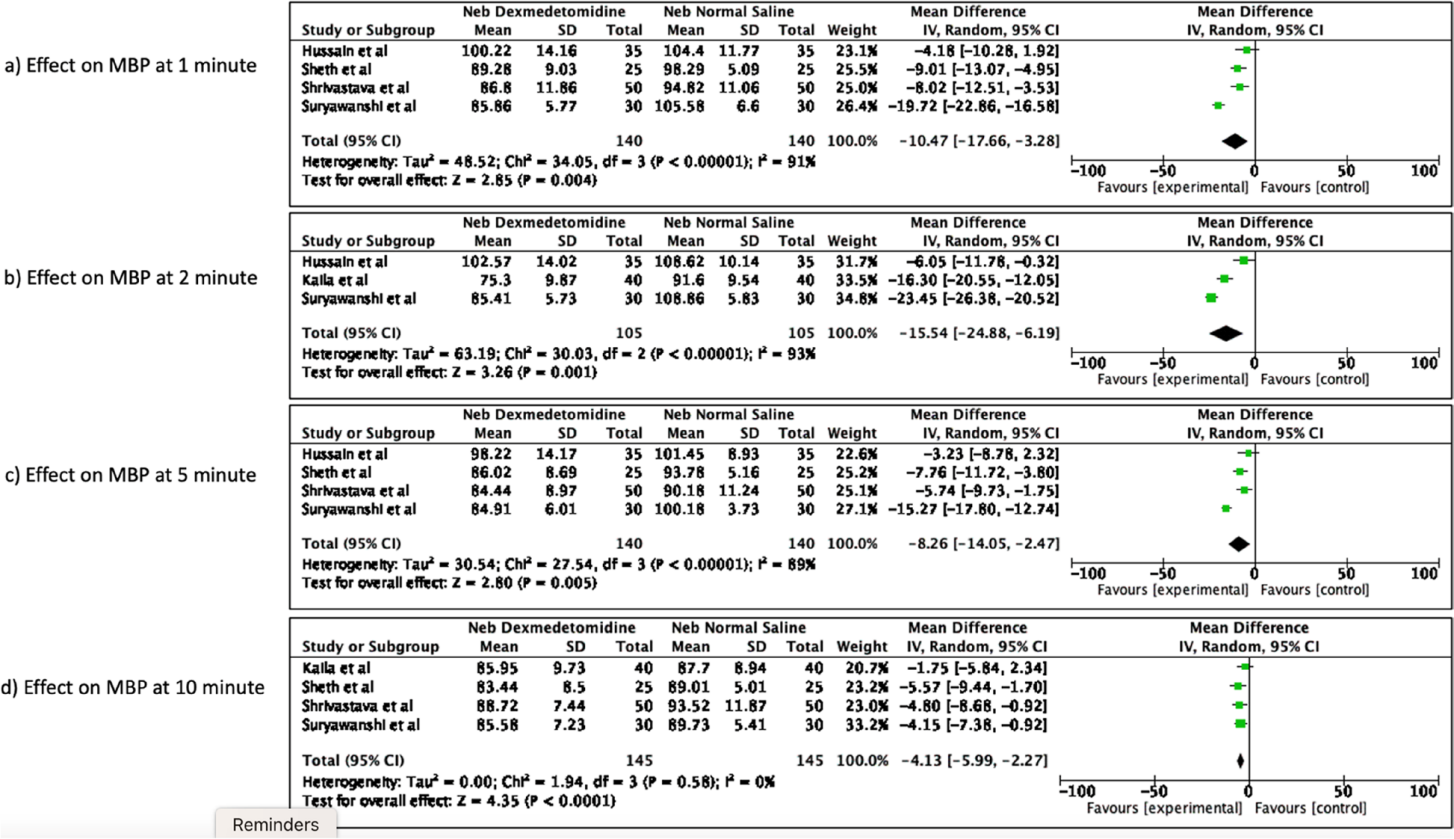
Effect of nebulized dexmedetomidine on MBP

Just like DBP, the significant reduction in MBP was seen in nebulized dexmedetomidine group as compared to normal saline group at all the measured time points after endotracheal intubation from 1 min till 10 min [mean difference -10.47 (95% CI -17.66 to -3.28), *p* = 0.004, I^2^ of 91% at 1 min; mean difference -15.54 (95% CI -24.88 to -6.19), *p* = 0.001, I^2^ = 93% at 2 min; mean difference -8.26 (95% CI -14.05 to -2.47), *p* = 0.005, I^2^ = 89% at 5 min; mean difference -4.13 (95% CI -5.99 to -2.27), *p* < 0.0001, I^2^ = 0% at 10 min] post-intubation. The forest plots are available as Fig. 5.

##### Safety assessment

The included studies did not report any adverse effects like intraoperative bradycardia or hypotension with use of nebulized dexmedetomidine, unlike intravenous dexmedetomidine during intubation. Post-operative nausea and vomiting was reported by only one study, Misra et al. in 3/57 patients (5.26%) in dexmedetomidine group and 1/59 (1.69%) in normal saline group (Table 1).

### Risk of bias assessment

**Fig. 6:**
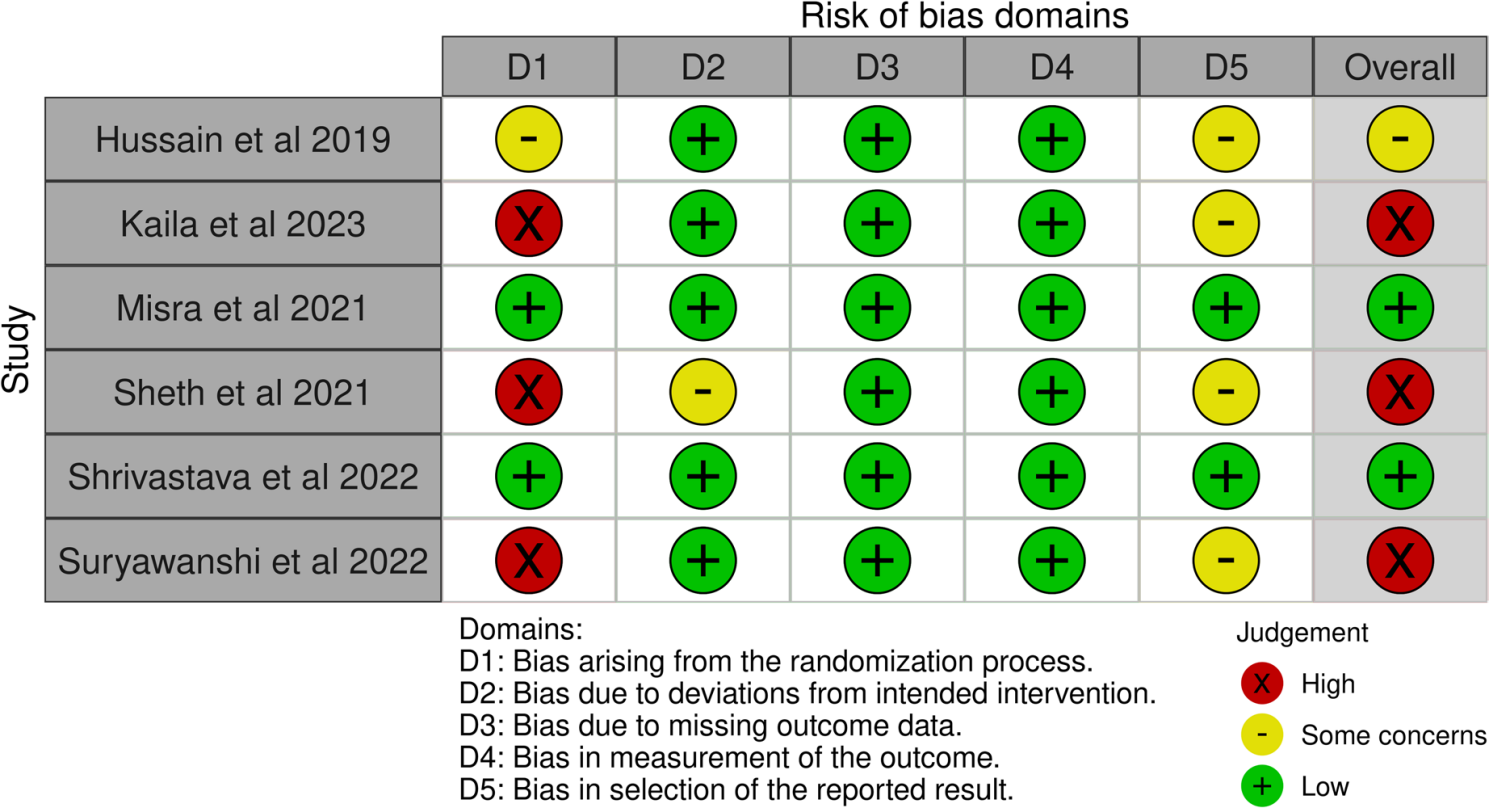
RoB2 assessment in various domains

According to RoB2 tool, three out of six included studies had a high risk of bias (Fig. 6). All the included studies described the method of randomization and were considered low risk except Sheth et al. and Kaila et al. which although mentioned that patients were randomized into two groups but the method of randomization was not mentioned [17, 20]. Suryawanshi et al. describe lottery method to randomly allocate the groups which is not considered the recommended method of randomization [18]. Information about allocation concealment was only mentioned by Misra et al. and Shrivastava et al. and were considered as low risk, rest all studies were considered at either high or unclear risk for bias arising from the randomization process [12, 19]. The blinding of the participants and personnel was done in all the studies (double blinded), except Sheth et al. which was judged to be at unclear risk of bias due to deviation from the intended interventions (Fig. 4) [17]. Except for Misra et al. and Shrivastava et al., none of the included studies provided trial registration number and were considered to have unclear risk of bias in selection of reported results [12, 19].

### Sensitivity analysis

Sensitivity analysis was performed after excluding Hussain et al. as this study differed from other RCTs in terms of dose of nebulized dexmedetomidine [16]. The dose used was 2 μg /kg dexmedetomidine whereas in other studies the dose used was 1 μg/kg dexmedetomidine, without any other methodological differences. Exclusion was not associated with any major change in heterogeneity or the effect estimate. The results of sensitivity analysis have been summarized in Supplementary files 2 and 3.

### Publication bias assessment

Funnel plots was plotted to assess for publication bias for the primary outcome effect on HR at 1 min for the studies included (Supplementary file 4). The publication bias is towards the studies with reduction in the hemodynamic response with nebulized dexmedetomidine.

### Strength of evidence

GRADE assessment revealed very low quality evidence for effect of nebulized dexmedetomidine in reducing HR and SBP response to endotracheal intubation at 1, 2 and 5 min (Table 2).

**Table 2:** GRADE assessment. Question: Is nebulized dexmedetomidine effective in blunting hemodynamic response to endotracheal intubation? Setting: Adult patients undergoing surgeries under general anaesthesia

**Certainty assessment**							**№ of patients**		**Effect**		**Certainty**	**Importance**
**№ of studies**	**Study design**	**Risk of bias**	**Inconsistency**	**Indirectness**	**Imprecision**	**Other considerations**	**dexmedetomidine nebulization**	**placebo or no intervention**	**Relative (95% CI)**	**Absolute (95% CI)**		
HR at 1 min (assessed with: beats per minute)												
**5**	randomised trials	Serious^a^	serious	not serious	not serious	publication bias strongly suspected	200	200	-	MD **8.59 beats/min lower** (16.42 lower to 0.75 lower)	⨁◯◯◯ Very low	There is very low quality evidence that preoperative nebulized dexmedetomidine compared is effective in reducing HR response to endotracheal intubation at 1 min.
HR at 2 min (assessed with: beats per minute)												
**4**	randomised trials	Serious^a^	serious^b^	not serious	not serious	publication bias strongly suspected	165	165	-	MD 13.48 **beats/min lower** (21.03 lower to 5.94 lower)	⨁◯◯◯ Very low	There is very low quality evidence that preoperative nebulized dexmedetomidine compared is effective in reducing HR response to endotracheal intubation at 2 min.
HR at 5 min (assessed with: beats/minute)												
**5**	randomised trials	Serious^a^	serious^b^	not serious	not serious	publication bias strongly suspected	200	200	-	MD **7.16 beats/min lower** (12.49 lower to 1.83 lower)	⨁◯◯◯ Very low	There is very low quality evidence that preoperative nebulized dexmedetomidine compared is effective in reducing HR response to endotracheal intubation at 5 min.
SBP at 1 min (assessed with: mmHg)												
**5**	randomised trials	Serious^a^	serious^b^	not serious	not serious	publication bias strongly suspected	200	200	-	MD **12.48 mmHg lower** (19.85 lower to 5.1 lower)	⨁◯◯◯ Very low	There is very low quality evidence that preoperative nebulized dexmedetomidine compared is effective in reducing SBP response to endotracheal intubation at 1 min.
SBP at 2 min (assessed with: mmHg)												
**4**	randomised trials	Serious^a^	serious^b^	not serious	not serious	publication bias strongly suspected	165	165	-	MD **21 mmHg lower** (30.41 lower to 11.6 lower)	⨁◯◯◯ Very low	There is very low quality evidence that preoperative nebulized dexmedetomidine compared is effective in reducing SBP response to endotracheal intubation at 2 min.
SBP at 5 min (assessed with: mmHg)												
**5**	randomised trials	Serious^a^	serious^b^	not serious	not serious	publication bias strongly suspected	200	200	-	MD **9.25 mmHg lower** (14.99 lower to 3.51 lower)	⨁◯◯◯ Very low	There is very low quality evidence that preoperative nebulized dexmedetomidine compared is effective in reducing SBP response to endotracheal intubation at 5 min.

*CI* Confidence interval, *MD* Mean difference

^a^Multiple biases at individual study level

^b^High heterogeneity and variability in effect estimates

## Discussion

The findings of this SRMA suggests that premedication with dexmedetomidine nebulization significantly attenuates the hemodynamic response to laryngoscopy and ETI in comparison to normal saline nebulization. Laryngoscopy and ETI are associated with sympathetic stimulation leading to various hemodynamic changes like tachycardia, hypertension and increase in intracranial pressure which could be life threatening in patients with underlying cardio- or cerebro-vascular comorbidities [24]. The nebulized dexmedetomidine was found to reduce the mean HR as compared to normal saline at all the time points included (1, 2, 3, 4, 5, 6, 8, 10 min). Similar reduction was seen in SBP, DBP and MBP. However, the heterogeneity was found to be high across the studies. In sync with our findings, a recent RCT also found nebulized dexmedetomidine to effectively blunt the pressor response to laryngoscopy and ETI, better than that of nebulized fentanyl and equivalent to that of nebulized magnesium sulphate [25]. Dexmedetomidine’s highly-selective agonistic action on presynaptic α2-adrenergic receptors and subsequent inhibition of norepinephrine release from the locus coeruleus has been hypothesized as the most putative mechanism for its hemodynamic stress response attenuating action [26]. Intravenous dexmedetomidine also attenuates the hemodynamic responses to laryngoscopy and ETI but is associated with risk of bradycardia, hypotension and cardiac arrests [4, 9, 10, 14, 27, 28]. De Cassai et al. in a recent SRMA of 99 RCTs involving 6833 patients found significant bradycardia in one out of every 12 patients [4]. Nebulized dexmedetomidine provides an alternative route and was found to be devoid of these intra-operative adverse effects in this SRMA. Our findings corroborate with similar findings by other authors [14]. The heart rate safety profile of nebulized versus IV dexmedetomidine might be advantageous in patients with low baseline HR such as those on pre-operative beta-blocker therapy [13, 14]. Also, nebulized dexmedetomidine causes less postoperative sedation than IV dexmedetomidine which may be beneficial in resource-poor settings with inadequate postoperative monitoring facilities and in patients with obstructive sleep apnoea or chronic obstructive pulmonary disease in whom postoperative sedation might be detrimental [14].

Other potential benefits of nebulized dexmedetomidine, as observed in included studies in this SRMA, involved reduction in the induction dose of propofol, intraoperative requirement of opioids and halogenated anaesthetics and incidence of postoperative sore-throat. However, low number of studies precluded meta-analysis of these outcome. Some of these advantages might be attributed to sedative and analgesic action of dexmedetomidine by virtue of its α2-agonistic action on the locus coeruleus [3]. Its dose-sparing effect on opioid and anaesthetic requirements have been shown to be comparable with intravenous dexmedetomidine [14]. Its short half-life and elimination life and easy acceptability in addition to its ability to provide a calm and sedated patient at induction, lower anaesthetic and analgesic requirements and devoid of adverse effects make nebulized dexmedetomidine an ideal premedication agent [13, 29]. The sedative action of nebulized dexmedetomidine is particularly advantageous in pediatric patients in whom it has been shown to reduce separation anxiety, recovery time, postoperative agitation, postoperative nausea and vomiting and improve mask acceptance; with nebulized dexmedetomidine shown to be better compared with nebulized ketamine and midazolam [3, 29, 30]. Another recent systematic review of 10 RCTs including 1233 patients established sedative efficacy of nebulized dexmedetomidine in pediatric patients undergoing medical examination or surgery [3]. Nebulized dexmedetomidine has been shown to ease and improve acceptability of IV cannulation; difficult in pediatric population owing to small veins and physical agitation [8, 31–33]. Postoperative sore throat is a common adverse effect after laryngoscopy and ETI and is associated with patient discomfort and dis-satisfaction after GA [34]. Congruent with our findings, others have also shown nebulized dexmedetomidine to reduce postoperative sore throat, better than that of IV dexmedetomidine [14]. This might be attributed to its anti-inflammatory action [14].

Sensitivity analysis by excluding study using a higher dose of nebulized dexmedetomidine (2 μg/kg) did not reveal any major impact on the pooled effect estimate, suggesting against a dose-response effect. However, only one of the included RCT used a higher dose of dexmedetomidine precluding any conclusion on the dose-response effect of nebulized dexmedetomidine on the hemodynamic stress response. This warrants future studies comparing different doses of dexmedetomidine to confirm or refute any dose-response effect.

### Limitations and strengths

This SRMA had few limitations. Exclusion of grey literature search and non-English studies might have led to missing out relevant articles. However, a comprehensive search of six (both uni-and multi-disciplinary) most relevant databases including google scholar (cataloguing both academic and grey literature) along with reference list and citation tracking were adopted to ensure that SR findings are informed by the best available evidence on the topic.

Secondly, only one-third of the included studies were at low risk of bias which reduced our certainty in the strength of evidence. This not only enable readers to view the available evidence in light of its quality but also provides useful insights for future triallists to improve the design and conduct of future RCTs to reduce the risk of biases identified in this SRMA.

Another limitation worth considering is the high heterogeneity observed in the effect estimates. A high I^2^ value, for e.g. of 92% for HR at 1 min reflect that 92% of variance in the observed effect is due to variance in true effect and only 8% is due to variance in the sampling error. This was despite the study sample across the studies being quite homogenous with respect to the age, ASA physical status and surgeries as outlined in 3.2. Except for Hussain et al., all others used 1 μg/kg as the dose of dexmedetomidine. A sensitivity analysis performed after excluding Hussain et al. in fact increased the heterogeneity for HR at 1 min (I^2^ from 92 to 94%). The main source of clinical heterogeneity evident among the studies was the use of anti-emetics, anti-cholinergic and benzodiazepines as premedication, some of which (e.g. midazolam) have themselves been shown to attenuate hemodynamic response to ETI [35].

To the best of authors knowledge, this is the first SRMA to systematically and comprehensively evaluate efficacy and safety of dexmedetomidine nebulization for attenuating hemodynamic response to ETI. The strength of this SRMA lies in its transparent and rigorous methodology to identify, collate, appraise and synthesize the available evidence informing the review topic. Key review decisions were made in consultation with the Cochrane Handbook for Systematic Review of Interventions and the project advisory group comprising of both the subject and methodology experts [36]. Two independent reviewer with arbitration and audit process was adopted at screening, data extraction, meta-analysis, risk of bias and GRADE assessment.

## Conclusions

Preoperative dexmedetomidine nebulization significantly reduces HR and BP response to laryngoscopy and ETI without any risk of adverse effects like bradycardia and hypotension. However, the strength of evidence is very low and warrants future properly designed and conducted RCTs to improve generalizability and strength of evidence. Future studies should also focus upon comparing different routes of dexmedetomidine administration and different doses of nebulized dexmedetomidine to establish a dose-response effect.

### Supplementary Information

**Additional file 1: Supplementary Table I.** Database Search Strategies. **Supplementary file 2.** Sensitivity analysis of Heart Rate. **Supplementary file 3.** Sensitivity analysis of Systolic Blood Pressure. **Supplementary file 4.** Funnel Plot of HR at 1 min.

## Data Availability

All data generated or analysed during this study are included in this published article [and its supplementary information files].

## Abbreviations

BP: Blood Pressure
DBP: Diastolic blood pressure
ETI: Endotracheal intubation
HR: Heart Rate
HT: Hemanthkumar Tamilchelvan
MBP: Mean blood pressure
MG: Mayank Gupta
PRISMA: Preferred Reporting Items for Systematic reviews and Meta-analysis
PROSPERO: International Prospective Register of Systematic Reviews
RCT: Randomized Controlled Trial
RoB: Risk of Bias
SBP: Systolic blood pressure
SRMA: Systematic review and meta-analysis
UJ: Udita Joshi
