# Supplementary material for: Nebulized dexmedetomidine for attenuating hemodynamic response to laryngoscopy and endotracheal intubation in adult patients undergoing surgeries under general anaesthesia: a systematic review and meta-analysis of randomized controlled trials
Source: BMC Anesthesiol. 2023 Dec 11;23:406. doi: 10.1186/s12871-023-02366-9 (PMC10712167; doi:10.1186/s12871-023-02366-9)

## Supplementary Table I: Database Search Strategies

### 1.1. PubMed, Searched on 31/03/2013

| S.No. | Search String | Hits |
| --- | --- | --- |
| 1 | "Laryngoscopy"[MeSH Terms] OR "intubation, intratracheal"[MeSH Terms] OR "Rapid Sequence Induction and Intubation"[MeSH Terms] | [52,081](https://pubmed.ncbi.nlm.nih.gov/?term=%22Laryngoscopy%22%5BMeSH+Terms%5D+OR+%22intubation%2C+intratracheal%22%5BMeSH+Terms%5D+OR+%22Rapid+Sequence+Induction+and+Intubation%22%5BMeSH+Terms%5D&sort=pubdate) |
| 2 | "laryngoscop*"[Title/Abstract] OR "endotracheal intubat*"[Title/Abstract] OR "intratracheal intubat*"[Title/Abstract] OR "intra tracheal intubat*"[Title/Abstract] OR "tracheal intubat*"[Title/Abstract] | [33,338](https://pubmed.ncbi.nlm.nih.gov/?term=%28laryngoscop%2A%5BTitle%2FAbstract%5D+OR+%22endotracheal+intubat%2A%22%5BTitle%2FAbstract%5D+OR+%22intratracheal+intubat%2A%22%5BTitle%2FAbstract%5D+OR+%22intra+tracheal+intubat%2A%22%5BTitle%2FAbstract%5D+OR+%22tracheal+intubat%2A%22%5BTitle%2FAbstract%5D%29&sort=pubdate) |
| 3 | #1 OR #2 | [69,590](https://pubmed.ncbi.nlm.nih.gov/?term=%28%22Laryngoscopy%22%5BMeSH+Terms%5D+OR+%22intubation%2C+intratracheal%22%5BMeSH+Terms%5D+OR+%22Rapid+Sequence+Induction+and+Intubation%22%5BMeSH+Terms%5D%29+OR+%28%28laryngoscop%2A%5BTitle%2FAbstract%5D+OR+%22endotracheal+intubat%2A%22%5BTitle%2FAbstract%5D+OR+%22intratracheal+intubat%2A%22%5BTitle%2FAbstract%5D+OR+%22intra+tracheal+intubat%2A%22%5BTitle%2FAbstract%5D+OR+%22tracheal+intubat%2A%22%5BTitle%2FAbstract%5D%29%29&sort=pubdate) |
| 4 | "Dexmedetomidine"[MeSH Terms] | [5,225](https://pubmed.ncbi.nlm.nih.gov/?term=%22Dexmedetomidine%22%5BMeSH+Terms%5D&sort=pubdate) |
| 5 | (MPV-1440 OR "MPV 1440" OR MPV1440 OR dex OR dexmed* OR dexdomitor OR dexdor OR cepedex OR precedex OR primadex OR sedadex OR sileo OR dextomid OR dexem OR dexmeto) [All Fields] | [19,548](https://pubmed.ncbi.nlm.nih.gov/?term=%28MPV-1440+OR+%22MPV+1440%22+OR+MPV1440+OR+dex+OR+dexmed%2A+OR+dexdomitor+OR+dexdor+OR+cepedex+OR+precedex+OR+primadex+OR+sedadex+OR+sileo+OR+dextomid+OR+dexem+OR+dexmeto%29&sort=pubdate) |
| 6 | #4 or #5 | [19,548](https://pubmed.ncbi.nlm.nih.gov/?term=%28%22Dexmedetomidine%22%5BMeSH+Terms%5D%29+OR+%28%28MPV-1440+OR+%22MPV+1440%22+OR+MPV1440+OR+dex+OR+dexmed%2A+OR+dexdomitor+OR+dexdor+OR+cepedex+OR+precedex+OR+primadex+OR+sedadex+OR+sileo+OR+dextomid+OR+dexem+OR+dexmeto%29%29&sort=pubdate) |
| 7 | #3 AND #6 | [456](https://pubmed.ncbi.nlm.nih.gov/?term=%28%28%22Laryngoscopy%22%5BMeSH+Terms%5D+OR+%22intubation%2C+intratracheal%22%5BMeSH+Terms%5D+OR+%22Rapid+Sequence+Induction+and+Intubation%22%5BMeSH+Terms%5D%29+OR+%28%28laryngoscop%2A%5BTitle%2FAbstract%5D+OR+%22endotracheal+intubat%2A%22%5BTitle%2FAbstract%5D+OR+%22intratracheal+intubat%2A%22%5BTitle%2FAbstract%5D+OR+%22intra+tracheal+intubat%2A%22%5BTitle%2FAbstract%5D+OR+%22tracheal+intubat%2A%22%5BTitle%2FAbstract%5D%29%29%29+AND+%28%28%22Dexmedetomidine%22%5BMeSH+Terms%5D%29+OR+%28%28MPV-1440+OR+%22MPV+1440%22+OR+MPV1440+OR+dex+OR+dexmed%2A+OR+dexdomitor+OR+dexdor+OR+cepedex+OR+precedex+OR+primadex+OR+sedadex+OR+sileo+OR+dextomid+OR+dexem+OR+dexmeto%29%29%29&sort=pubdate) |
| 8 | Search: "Randomized Controlled Trial"[Publication Type] OR "Randomized Controlled Trials as Topic"[MeSH Terms] OR "Controlled Clinical Trial"[Publication Type] OR "Clinical Trial"[Publication Type] OR "Clinical Trials as Topic"[MeSH Terms] | [1,266,924](https://pubmed.ncbi.nlm.nih.gov/?term=%22Randomized+Controlled+Trial%22%5BPublication+Type%5D+OR+%22Randomized+Controlled+Trials+as+Topic%22%5BMeSH+Terms%5D+OR+%22Controlled+Clinical+Trial%22%5BPublication+Type%5D+OR+%22Clinical+Trial%22%5BPublication+Type%5D+OR+%22Clinical+Trials+as+Topic%22%5BMeSH+Terms%5D&sort=pubdate) |
| 9 | "randomized"[Title/Abstract] OR "placebo"[Title/Abstract] OR control[Title/Abstract] OR "random*"[Title/Abstract] OR "trial"[Title/Abstract] | [4,458,011](https://pubmed.ncbi.nlm.nih.gov/?term=%22randomized%22%5BTitle%2FAbstract%5D+OR+%22placebo%22%5BTitle%2FAbstract%5D+OR+control%5BTitle%2FAbstract%5D+OR+%22random%2A%22%5BTitle%2FAbstract%5D+OR+%22trial%22%5BTitle%2FAbstract%5D&sort=pubdate) |
| 10 | #8 OR #9 | [4,918,932](https://pubmed.ncbi.nlm.nih.gov/?term=%28%22Randomized+Controlled+Trial%22%5BPublication+Type%5D+OR+%22Randomized+Controlled+Trials+as+Topic%22%5BMeSH+Terms%5D+OR+%22Controlled+Clinical+Trial%22%5BPublication+Type%5D+OR+%22Clinical+Trial%22%5BPublication+Type%5D+OR+%22Clinical+Trials+as+Topic%22%5BMeSH+Terms%5D%29+OR+%28%22randomized%22%5BTitle%2FAbstract%5D+OR+%22placebo%22%5BTitle%2FAbstract%5D+OR+control%5BTitle%2FAbstract%5D+OR+%22random%2A%22%5BTitle%2FAbstract%5D+OR+%22trial%22%5BTitle%2FAbstract%5D%29&sort=pubdate) |
| 11 | #7 AND #10 | [281](https://pubmed.ncbi.nlm.nih.gov/?term=%28%28%28%22Laryngoscopy%22%5BMeSH+Terms%5D+OR+%22intubation%2C+intratracheal%22%5BMeSH+Terms%5D+OR+%22Rapid+Sequence+Induction+and+Intubation%22%5BMeSH+Terms%5D%29+OR+%28%28laryngoscop%2A%5BTitle%2FAbstract%5D+OR+%22endotracheal+intubat%2A%22%5BTitle%2FAbstract%5D+OR+%22intratracheal+intubat%2A%22%5BTitle%2FAbstract%5D+OR+%22intra+tracheal+intubat%2A%22%5BTitle%2FAbstract%5D+OR+%22tracheal+intubat%2A%22%5BTitle%2FAbstract%5D%29%29%29+AND+%28%28%22Dexmedetomidine%22%5BMeSH+Terms%5D%29+OR+%28%28MPV-1440+OR+%22MPV+1440%22+OR+MPV1440+OR+dex+OR+dexmed%2A+OR+dexdomitor+OR+dexdor+OR+cepedex+OR+precedex+OR+primadex+OR+sedadex+OR+sileo+OR+dextomid+OR+dexem+OR+dexmeto%29%29%29%29+AND+%28%28%22Randomized+Controlled+Trial%22%5BPublication+Type%5D+OR+%22Randomized+Controlled+Trials+as+Topic%22%5BMeSH+Terms%5D+OR+%22Controlled+Clinical+Trial%22%5BPublication+Type%5D+OR+%22Clinical+Trial%22%5BPublication+Type%5D+OR+%22Clinical+Trials+as+Topic%22%5BMeSH+Terms%5D%29+OR+%28%22randomized%22%5BTitle%2FAbstract%5D+OR+%22placebo%22%5BTitle%2FAbstract%5D+OR+control%5BTitle%2FAbstract%5D+OR+%22random%2A%22%5BTitle%2FAbstract%5D+OR+%22trial%22%5BTitle%2FAbstract%5D%29%29&sort=pubdate) |
| 12 | Filter: English | [264](https://pubmed.ncbi.nlm.nih.gov/?term=%28%28%28%22Laryngoscopy%22%5BMeSH+Terms%5D+OR+%22intubation%2C+intratracheal%22%5BMeSH+Terms%5D+OR+%22Rapid+Sequence+Induction+and+Intubation%22%5BMeSH+Terms%5D%29+OR+%28%28laryngoscop%2A%5BTitle%2FAbstract%5D+OR+%22endotracheal+intubat%2A%22%5BTitle%2FAbstract%5D+OR+%22intratracheal+intubat%2A%22%5BTitle%2FAbstract%5D+OR+%22intra+tracheal+intubat%2A%22%5BTitle%2FAbstract%5D+OR+%22tracheal+intubat%2A%22%5BTitle%2FAbstract%5D%29%29%29+AND+%28%28%22Dexmedetomidine%22%5BMeSH+Terms%5D%29+OR+%28%28MPV-1440+OR+%22MPV+1440%22+OR+MPV1440+OR+dex+OR+dexmed%2A+OR+dexdomitor+OR+dexdor+OR+cepedex+OR+precedex+OR+primadex+OR+sedadex+OR+sileo+OR+dextomid+OR+dexem+OR+dexmeto%29%29%29%29+AND+%28%28%22Randomized+Controlled+Trial%22%5BPublication+Type%5D+OR+%22Randomized+Controlled+Trials+as+Topic%22%5BMeSH+Terms%5D+OR+%22Controlled+Clinical+Trial%22%5BPublication+Type%5D+OR+%22Clinical+Trial%22%5BPublication+Type%5D+OR+%22Clinical+Trials+as+Topic%22%5BMeSH+Terms%5D%29+OR+%28%22randomized%22%5BTitle%2FAbstract%5D+OR+%22placebo%22%5BTitle%2FAbstract%5D+OR+control%5BTitle%2FAbstract%5D+OR+%22random%2A%22%5BTitle%2FAbstract%5D+OR+%22trial%22%5BTitle%2FAbstract%5D%29%29&filter=lang.english&sort=pubdate) |
| 13 | Filter: Human | [143](https://pubmed.ncbi.nlm.nih.gov/?term=%28%28%28%22Laryngoscopy%22%5BMeSH+Terms%5D+OR+%22intubation%2C+intratracheal%22%5BMeSH+Terms%5D+OR+%22Rapid+Sequence+Induction+and+Intubation%22%5BMeSH+Terms%5D%29+OR+%28%28laryngoscop%2A%5BTitle%2FAbstract%5D+OR+%22endotracheal+intubat%2A%22%5BTitle%2FAbstract%5D+OR+%22intratracheal+intubat%2A%22%5BTitle%2FAbstract%5D+OR+%22intra+tracheal+intubat%2A%22%5BTitle%2FAbstract%5D+OR+%22tracheal+intubat%2A%22%5BTitle%2FAbstract%5D%29%29%29+AND+%28%28%22Dexmedetomidine%22%5BMeSH+Terms%5D%29+OR+%28%28MPV-1440+OR+%22MPV+1440%22+OR+MPV1440+OR+dex+OR+dexmed%2A+OR+dexdomitor+OR+dexdor+OR+cepedex+OR+precedex+OR+primadex+OR+sedadex+OR+sileo+OR+dextomid+OR+dexem+OR+dexmeto%29%29%29%29+AND+%28%28%22Randomized+Controlled+Trial%22%5BPublication+Type%5D+OR+%22Randomized+Controlled+Trials+as+Topic%22%5BMeSH+Terms%5D+OR+%22Controlled+Clinical+Trial%22%5BPublication+Type%5D+OR+%22Clinical+Trial%22%5BPublication+Type%5D+OR+%22Clinical+Trials+as+Topic%22%5BMeSH+Terms%5D%29+OR+%28%22randomized%22%5BTitle%2FAbstract%5D+OR+%22placebo%22%5BTitle%2FAbstract%5D+OR+control%5BTitle%2FAbstract%5D+OR+%22random%2A%22%5BTitle%2FAbstract%5D+OR+%22trial%22%5BTitle%2FAbstract%5D%29%29&filter=lang.english&filter=hum_ani.humans&sort=pubdate) |

### 1.2. Embase (OvidSP) 1947 to March 30 2023, searched on 31/03/2023

| # | Search String | Hits |
| --- | --- | --- |
| 1 | exp laryngoscopy/ | 29,503 |
| 2 | exp endotracheal intubation/ | 64,298 |
| 3 | laryngoscop*.mp. | 40,596 |
| 4 | (intubat* adj6 (tracheal or intratracheal or endotracheal)).mp. | 72,004 |
| 5 | 1 or 2 or 3 or 4 | 102,764 |
| 6 | exp dexmedetomidine/ | 17,913 |
| 7 | (MPV-1440 or MPV1440 or "MPV 1440" or dex or dexmed* or dexdomitor or dexdor or cepedex or precedex or primadex or sedadex or sileo or dextomid or dexem or dexmeto).mp. | 34,078 |
| 8 | 6 or 7 | 34,078 |
| 9 | 5 and 8 | 1,631 |
| 10 | exp randomized controlled trial/ | 781,854 |
| 11 | exp controlled clinical trial/ | 974,500 |
| 12 | exp clinical trial/ | 1,852,956 |
| 13 | (random* or placebo or control* or trial).tw. | 7,635,498 |
| 14 | ("randomized controlled trial" or "controlled clinical trial").tw. | 159,167 |
| 15 | 10 or 11 or 12 or 13 or 14 | 8,365,223 |
| 16 | 9 and 15 | 791 |
| 17 | limit 16 to (human and english language) | 632 |

### 1.3. Cochrane Library (all sections), searched via Wiley on 31/03/2013

| S.No. | Search string | Hits |
| --- | --- | --- |
| 1 | MeSH descriptor: [Laryngoscopy] explode all trees | 1442 |
| 2 | MeSH descriptor: [Intubation, Intratracheal] explode all trees | 5076 |
| 3 | (laryngoscop*):ti,ab,kw | 5306 |
| 4 | ((tracheal or intratracheal or endotracheal) NEXT intubat*):ti,ab,kw | 10163 |
| 5 | #1 or #2 or #3 or #4 | 14725 |
| 6 | MeSH descriptor: [Dexmedetomidine] explode all trees | 2483 |
| 7 | MPV-1440 or MPV1440 or "MPV 1440" or dex or dexmed* or dexdomitor or dexdor or cepedex or precedex or primadex or sedadex or sileo or dextomid or dexem or dexmeto | 9369 |
| 8 | #6 or #7 | 9369 |
| 9 | #5 and #8 | 733 |
| 10 | MeSH descriptor: [Randomized Controlled Trial] explode all trees | 25744 |
| 11 | MeSH descriptor: [Clinical Trial] explode all trees | 45359 |
| 12 | (random* or placebo or control* or trial):ti,ab,kw | 1567841 |
| 13 | ("randomized controlled trial" or "controlled clinical trial"):ti,ab,kw | 648247 |
| 14 | #10 or #11 or #12 or #13 | 1567841 |
| 15 | #9 and #14 | 687 |

### 1.4. Web of Science, searched via Clarivate on 31/03/2023

| S.No. | Search String | Hits |
| --- | --- | --- |
| 1 | TS=((laryngoscop* OR "endotracheal intubat*" OR "intratracheal intubat*" OR "intra tracheal intubat*" OR "tracheal intubat*")) | 32105 |
| 2 | TS=(mpv-1440 OR "mpv 1440" OR mpv1440 OR dex OR dexmed* OR dexdomitor OR dexdor OR cepedex OR precedex OR primadex OR sedadex OR sileo OR dextomid OR dexem OR dexmeto) | 28515 |
| 3 | TS=("randomized controlled trial" OR "controlled clinical trial" OR "clinical trial" OR random* OR placebo OR control OR trial*) | 9843165 |
| 4 | #1 AND #2 AND #3 | 291 |
| 5 | Limit to English language | 285 |

### 1.5. Scopus, searched via Elsevier on 31/03/2023

| S.No. | Search String | Hits  (R1:MG) |
| --- | --- | --- |
| 1 | TITLE-ABS-KEY((laryngoscop* OR "endotracheal intubat*" OR "intratracheal intubat*" OR "intra tracheal intubat*" OR "tracheal intubat*")) | 95238 |
| 2 | TITLE-ABS-KEY(mpv-1440 OR "mpv 1440" OR mpv1440 OR dex OR dexmed* OR dexdomitor OR dexdor OR cepedex OR precedex OR primadex OR sedadex OR sileo OR dextomid OR dexem OR dexmeto) | 34541 |
| 3 | TITLE-ABS-KEY("randomized controlled trial" OR "controlled clinical trial" OR "clinical trial" OR random* OR placebo OR control OR trial*) | 13309055 |
| 4 | #1 AND #2 AND #3 | 773 |
| 5 | Limit to English language | 713 |

###

### 1.6. Google Scholar, searched on 31/03/2023


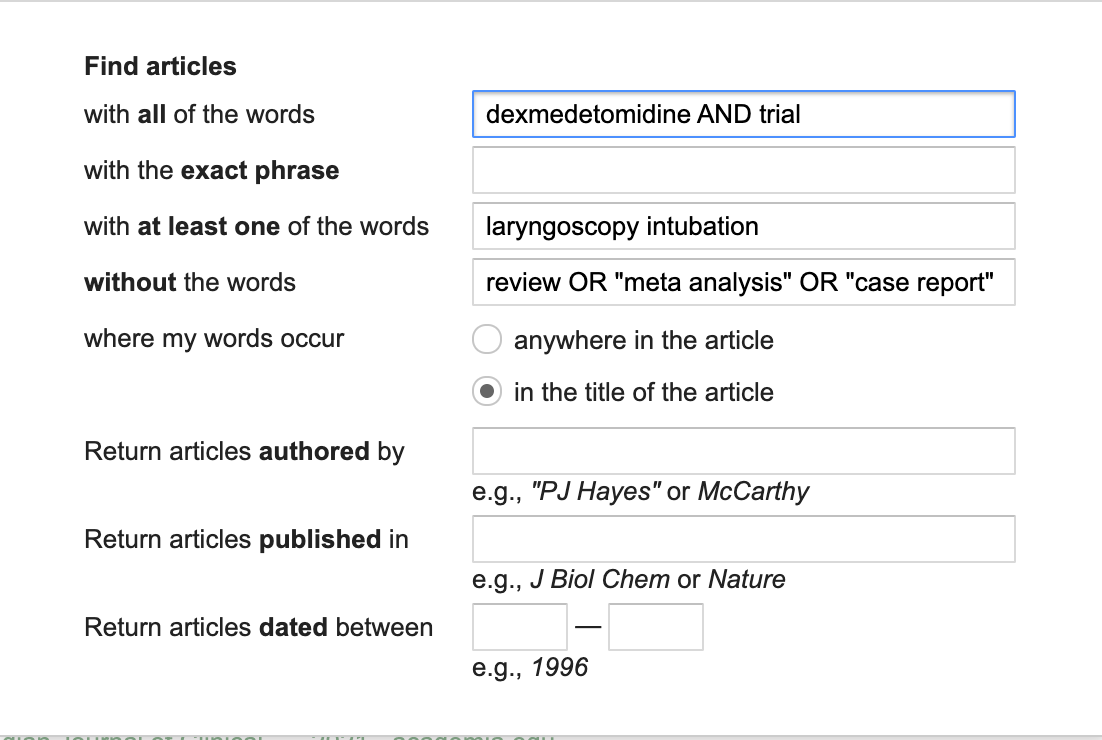


Supplementary file 2: Sensitivity analysis of Heart Rate


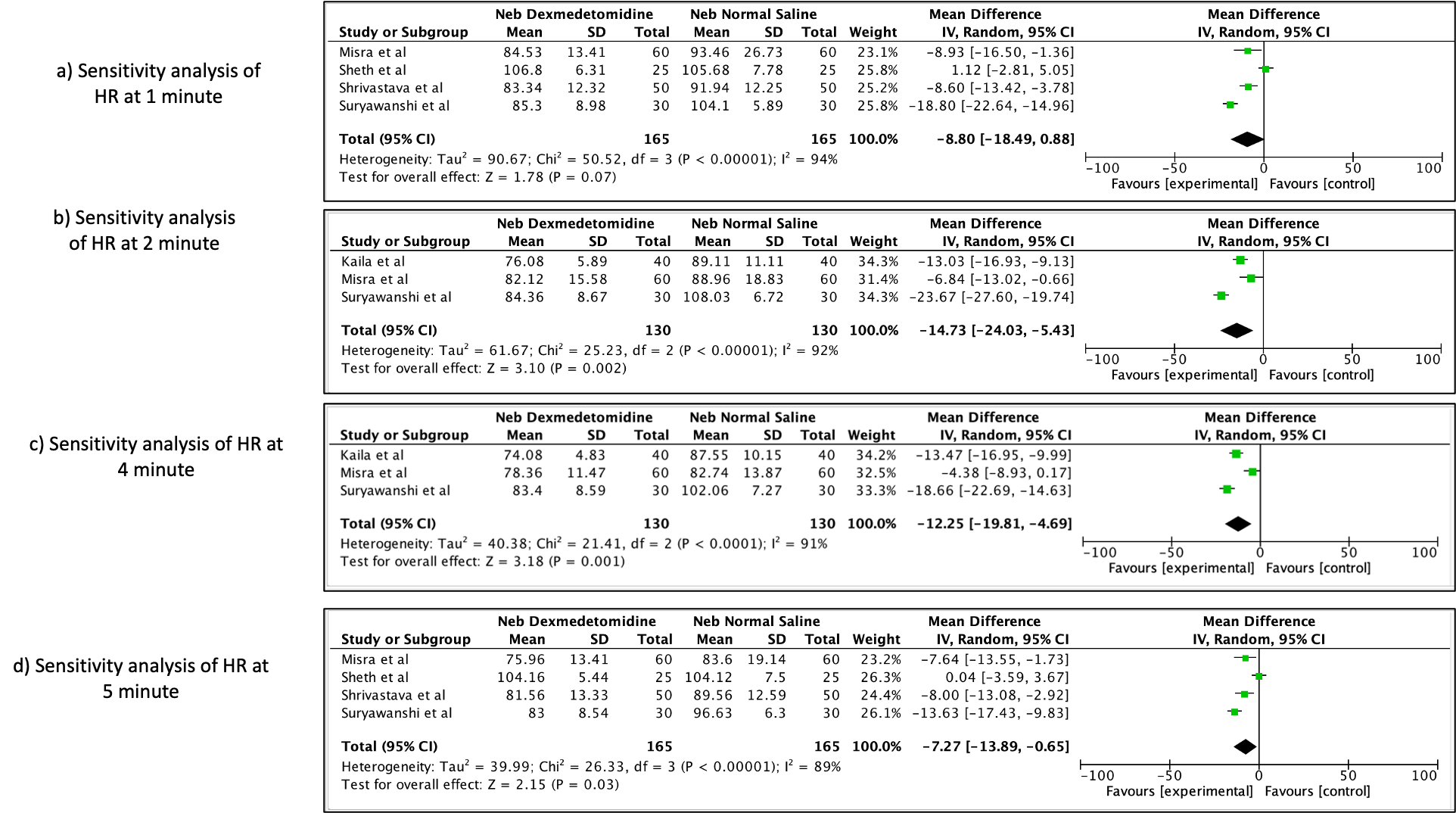


Supplementary file 3: Sensitivity analysis of Systolic Blood Pressure


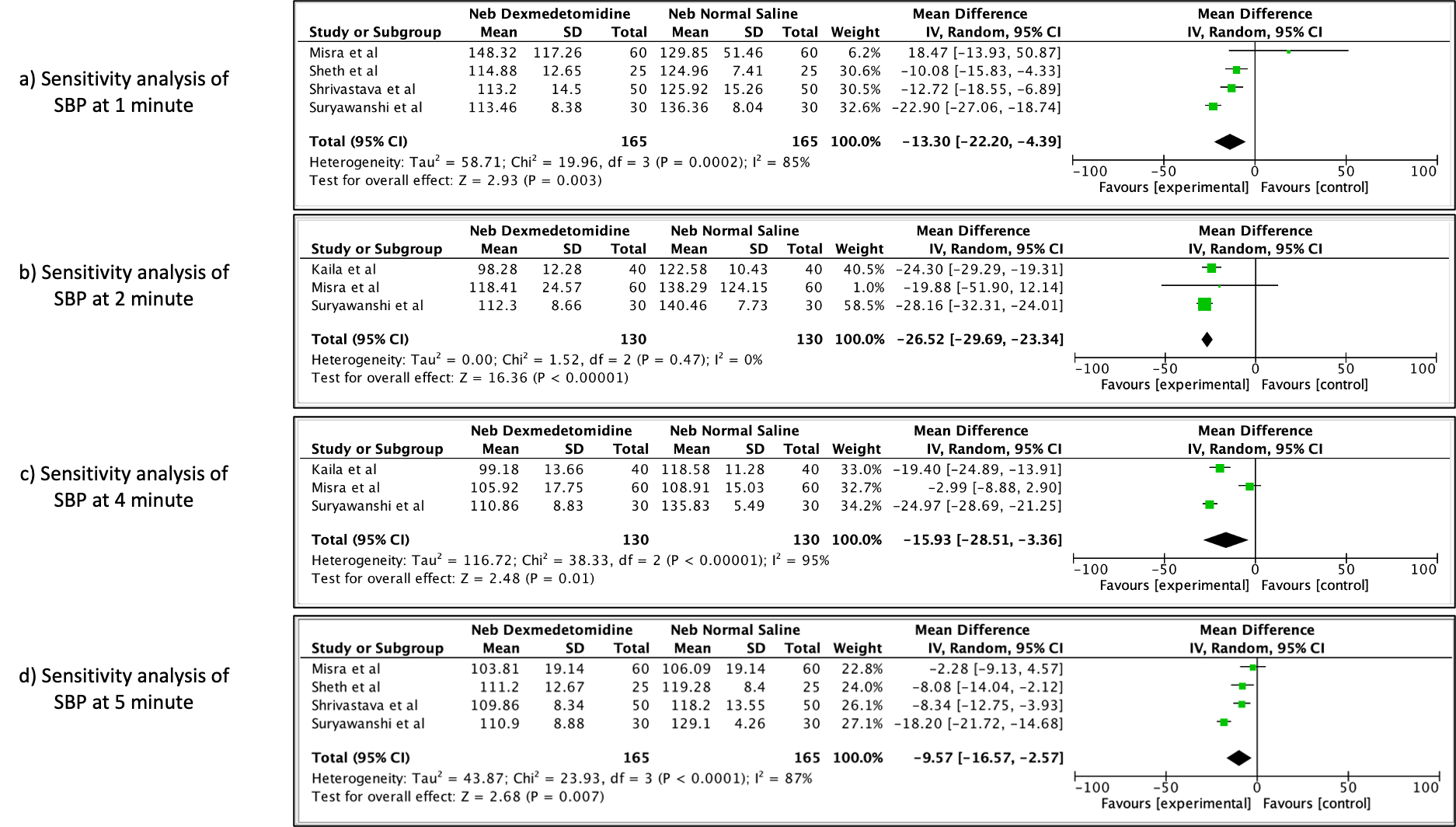


Supplementary file 4: Funnel Plot of HR at 1 minute


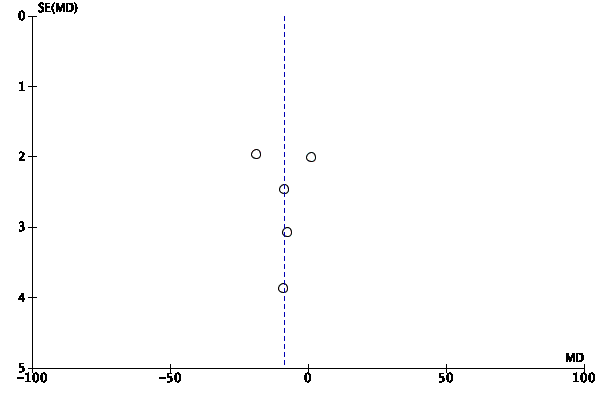

Supplement: Supplementary file 1 — Additional file 1: Supplementary Table I. Database Search Strategies. Supplementary file 2. Sensitivity analysis of Heart Rate. Supplementary file 3. Sensitivity analysis of Systolic Blood Pressure. Supplementary file 4. Funnel Plot of HR at 1 min. [file 12871_2023_2366_MOESM1_ESM.docx]
